# Unilateral cortical hyperintensity in diffusion-weighted MRI; New criteria for early sporadic Creutzfeldt-Jakob disease

**Published:** 2015-04-04

**Authors:** Nasim Tabrizi, Mahmoud Abedini

**Affiliations:** Department of Neurology, School of Medicine, Mazandaran University of Medical Sciences, Sari, Iran

**Keywords:** Creutzfeldt-Jakob Disease, Magnetic Resonance Imaging, Electroencephalography

Sporadic Creutzfeldt-Jakob disease (sCJD) is a rapidly progressive fatal prion disease. The proposed diagnostic criteria^1^^,^^2^ are not sufficiently helpful for diagnosis in early stages of the disorder.

A 69-year-old female was brought to our hospital with a history of 3 weeks left side hemiparesis and the progressive loss of speech and attention. She was awake and mute without any purposeful behavior. Left side hemiplegia, hyperreflexia and Babinski sign was also detected. Brain magnetic resonance imaging (MRI) revealed asymmetric diffuse gyriform hyperintensity in right cortical area and fine signal changes in right caudate and putamen in diffusion-weighted imaging (DWI) sequence without any significant involvement in left side and no signal abnormality in other sequences (Figure 1).

Laboratory tests, including blood count, glucose, renal, hepatic and thyroid function tests, electrolytes, sedimentation rate, B12 and folic acid levels were normal. Human T-lymphotropic virus 1 (HTLV1) and 2, human immunodeficiency virus (HIV) and paraneoplastic antibodies, anti-thyroid peroxidase, anti-thyroglobulin and venereal disease research laboratory tests were negative. A repeated MRI 7 days later revealed fine hyperintense signals in left inferior frontal, angular and postcentral gyri on DWI sequences, although the signal changes were still clearly asymmetric, and no abnormality was present in other sequences. At fifteenth day of admission, she experienced myoclonic jerks and 2 days later, the typical pattern of periodic sharp wave complexes was appeared in electroencephalography.

Pathological study of right frontal cortical biopsy disclosed neuronal loss and spongiform changes (Figure 2).

**Figure 1:**
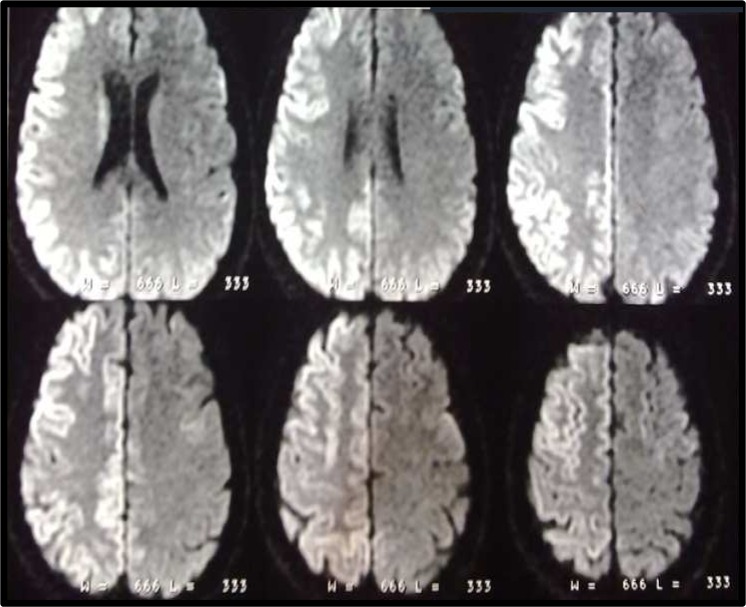
The brain magnetic resonance imaging diffusion (weighted imaging) shows right side diffuse cortical hyperintensity

**Figure 2:**
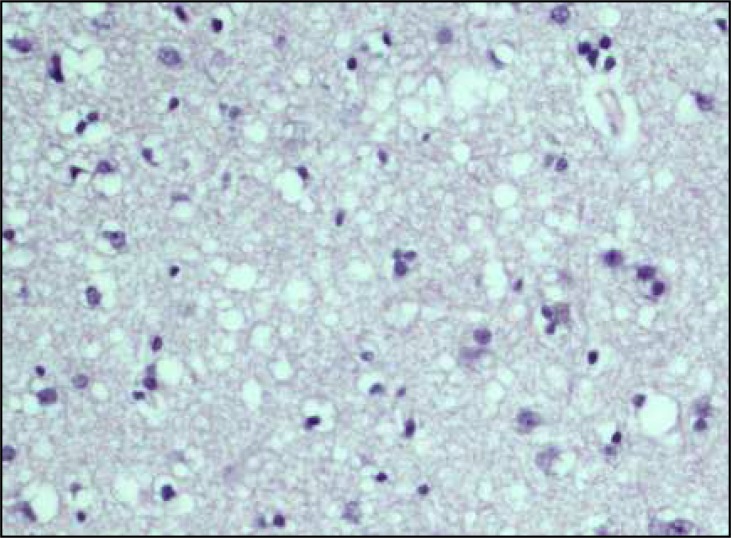
Spongiform changes in right frontal cortical biopsy (hematoxylin and eosin stain)

Based on World Health Organization (WHO) 1998 revised criteria,^1^ the diagnosis of probable sCJD confirms for our case and according to University of California, San Francisco (UCSF) 2005 and 2010 proposal of MRI criteria,^2^ the MRI is compatible with definite CJD. However, precentral and postcentral gyral involvement and the unilateral cortical hyperintensity merely in DWI sequences, even in late stages of the disease have made our case unique in the literature. Presence of unilateral gyral hyperintensity in DWI sequence should be considered as early imaging criteria for sCJD.

## Notes:

**How to cite this article: **Tabrizi N, Abedini M. Unilateral cortical hyperintensity in diffusion-weighted MRI; New criteria for early sporadic Creutzfeldt-Jakob disease. Iran J Neurol 2015; 14(2): 108-109.
